# Preoperative ALBI grade predicts the outcomes in non-B non-C HCC patients undergoing primary curative resection

**DOI:** 10.1186/s12876-021-01944-w

**Published:** 2021-10-19

**Authors:** Yu-Chieh Tsai, Fai-Meng Sou, Yueh-Wei Liu, Yi-Ju Wu, Chee-Chien Yong, Ding-Wei Chen, Pao-Yuan Huang, Wei-Ru Cho, Ching-Hui Chuang, Chang-Chun Hsiao, Tsung-Hui Hu, Ming-Chao Tsai

**Affiliations:** 1 Division of Hepato-Gastroenterology, Department of Internal Medicine, Kaohsiung Chang Gung Memorial Hospital and Chang Gung University College of Medicine, 123 Ta Pei Road, Kaohsiung, 83301 Taiwan; 2 Liver Transplantation Center and Department of Surgery, Kaohsiung Chang Gung Memorial Hospital and Chang Gung University College of Medicine, Kaohsiung, Taiwan; 3 Center for Translational Research in Biomedical Sciences, Liver Transplantation Program and Department of Surgery, Kaohsiung Chang Gung Memorial Hospital and Chang Gung University College of Medicine, Kaohsiung, Taiwan; 4 Department of Nursing, Meiho University, Pingtung, Taiwan; 5 Graduate Institute of Clinical Medical Sciences, College of Medicine, Chang Gung University, Division of Pulmonary and Critical Care Medicine, Kaohsiung Chang Gung Memorial Hospital, Kaohsiung, Taiwan; 6 Graduate Institute of Clinical Medical Sciences, Chang Gung University College of Medicine, Kaohsiung, Taiwan

**Keywords:** ALBI, NBNC, Hepatocellular carcinoma, Resection, Recurrence

## Abstract

**Background:**

The albumin–bilirubin (ALBI) grade has been validated as a significant prognostic predictor for hepatocellular carcinoma (HCC). However, there is little information about the ALBI grade in patients with non-B non-C HCC (NBNC-HCC) receiving surgery.

**Aim:**

This study aimed to evaluate the prognostic significance of the ALBI grade in patients with NBNC-HCC after primary curative resection.

**Method:**

From January 2010 to April 2016, 2137 patients with HCC who received hepatectomy were screened for study eligibility. Finally, a total of 168 NBNC-HCC patients who received primary curative resection were analyzed. The impacts of the ALBI grade on disease-free survival (DFS) and overall survival (OS) were analyzed by multivariate analysis.

**Results:**

There were 66 (39.3%), 98 (58.3%), and 4 (2.4%) patients with an ALBI grade of I, II, and III, respectively. Patients with an ALBI grade II/III were older (*p* = 0.002), more likely to have hypoalbuminemia (*p* < 0.001), and more commonly had Child–Pugh class B (*p* = 0.009) than patients with an ALBI grade I. After a median follow-up of 76 months, 74 (44%) patients experienced recurrence, and 72 (42.9%) patients died. Multivariate analysis revealed that alpha-fetoprotein (AFP) > 200 ng/mL (*p* = 0.021), number of tumors (*p* = 0.001), and tumor stage (*p* = 0.007) were independent prognostic factors for DFS. Additionally, AFP > 200 ng/mL (*p* = 0.002), ALBI grade II/III (*p* = 0.002), and tumor stage (*p* < 0.001) were independent risk factors for poor OS.

**Conclusion:**

The preoperative ALBI grade can be used to predict mortality in patients with NBNC-HCC after primary curative resection.

**Supplementary Information:**

The online version contains supplementary material available at 10.1186/s12876-021-01944-w.

## Introduction

Hepatocellular carcinoma (HCC) is the fifth most common cancer worldwide and the second most frequent cause of cancer-related death [1, 2]. Hepatitis B virus (HBV) and hepatitis C virus (HCV) infection are the main causes of HCC [3]. In Taiwan, the major cause of HCC is HBV infection, followed by HCV infection, which is similar to what has been observed in many other Asian countries [4–6]. With the introduction of a universal HBV vaccination program for newborns and infants, development of antiviral therapy for HBV and HCV infection, and changes in lifestyle, the incidence of virus-related HCC has decreased over the last decade. However, the number of HCC patients with neither HBV nor HCV infection, also known as non-B, non-C HCC (NBNC-HCC), has been increasing annually and currently accounts for 11% of all HCC cases in Taiwan [7]. This has also been observed in South Korea and Japan [8, 9], which are HCV-endemic countries. Therefore, NBNC-HCC is becoming a significant subgroup of HCC in areas of East Asia, despite this area being endemic for chronic hepatitis B (CHB) and CHC and having a high incidence of viral-related HCC.

The clinicopathologic characteristics and prognosis are quite different from HCC caused by viral hepatitis and non-viral hepatitis (NBNC-HCC) [10, 11]. Based on the study by Xue et al*.* [10], HBV and HCV related-HCC has a higher proportion of vascular invasion, and patients with HBV-HCC were significantly younger than NBNC-HCC. Besides, the prognosis of HBV-HCC was also worse than that of NBNC-HCC. According to a Japanese national registry data from the study by Utsunomiya et al. [11], liver function in the HCV-HCC group was significantly worse than that in the HBV- and NBNC-HCC groups. Multivariate analysis revealed a significantly better RFS in the NBNC-HCC group. They concluded that patients with NBNC HCC had a significantly lower risk of tumor recurrence than those with HBV and HCV derived HCC. Although some studies have compared NBNC-HCC patients with virus-related HCC patients with inconsistent results, possibly due to differences in demographic and tumor factors, and the number of patients in the cohort may be insufficient.

Curative resection is the most effective treatment for HCC; it can contribute to survival benefit for patients with early-stage disease when liver transplantation is not immediately accessible [12]. However, hepatic functional reserve is critical due to cirrhosis progression [13]. Child–Pugh grade is the most widely used assessment method for hepatic functional reserve. The Child–Pugh grade takes into account albumin, PT/INR, ascites, and hepatic encephalopathy, but some of these factors are highly subjective, such as the severity of ascites and degree of hepatic encephalopathy, which may affect assessment ability [14, 15]. Many recent studies have demonstrated the utility of the albumin–bilirubin (ALBI) grade, which was first described in 2015 by Johnson et al. [16], for evaluating hepatic function and predicting the prognosis of patients with HCC following liver resection [17–20]. However, most studies enrolled patients with viral-related HCC, and there was little information regarding the impact of the ALBI grade in patients with NBNC-HCC after curative resection. Because the prevalence of nonviral HCC is increasing in Taiwan, it is a good time to evaluate the effect of the preoperative ALBI grade in predicting the outcome of patients with NBNC-HCC after primary curative hepatectomy. This study aimed to evaluate the prognostic relevance of the ALBI grade in patients with NBNC-HCC after primary curative resection.

## Patients and methods

### Ethics statement

This study complies with the standards of the Declaration of Helsinki and current ethical guidelines, and approval was obtained from the Ethics Committee of Chang Gung Memorial Hospital (IRB approval number: 201901103B0). The requirement for informed consent for this study was waived by the Institutional Review Board, and all the data were analyzed anonymously.

### Patients and methods

**Fig. 1:**
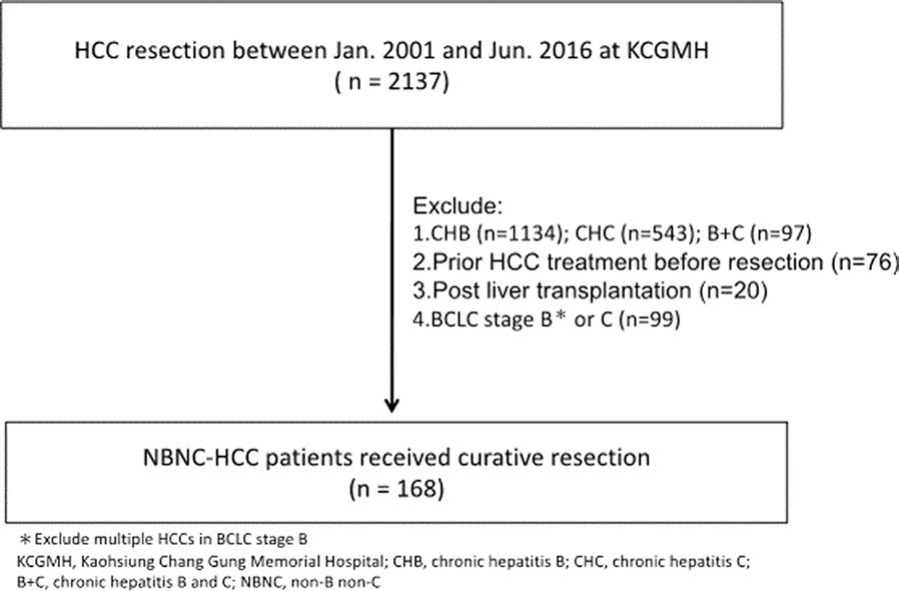
Schematic flowchart of the enrolment process

We reviewed a total of 2137 HCC patients who received surgical resection between January 2001 and April 2016 at the Kaohsiung Chang Gung Memorial Hospital. This hospital is a tertiary referral center that covers the southern part of Taiwan. The exclusion criteria were as follows: (a) serum hepatitis B surface antigen (HBsAg) positivity; (b) serum antibody hepatitis C (anti-HCV) positivity; (c) both serum HBsAg and anti-HCV positivity; (d) prior HCC treatment before surgical resection; (e) liver transplantation; (f) Barcelona Clinic Liver Cancer (BCLC) stage C; and (g) multiple HCCs in BCLC stage B. Finally, we enrolled 168 patients in this study (Fig. 1). The HCC diagnosis was based on the criteria of the American Association for the Study of Liver Disease (AASLD) and the European Association for the Study of the Liver (EASL) [21, 22] or confirmed by the histology results if they were available.

Information on patient demographics, serum biochemistry, and tumor burden was obtained through review of the medical records, and the diagnosis of cirrhosis was documented by the resected non-tumor pathologic report. Blood tests were performed within 1 week before resection. The ALBI score was calculated from the formula: ALBI score = (log_10_ bilirubin × 0.66) + (albumin ×  − 0.085), where the units of bilirubin and albumin were μmol/L and g/L, respectively. Patients were then stratified into three grades based on the ALBI score, as reported previously [16]: grade I, score ≤  − 2.60; grade II, score − 2.60 to ≤  − 1.39; and grade III, score >  − 1.39. Disease-free survival (DFS) was defined as the period from tumor removal by resection until the detection of recurrence. Overall survival (OS) was defined as the period from tumor removal by resection to death, last contact, or December 31, 2018.

### Statistical analysis

Statistical analyses were performed using SPSS 23.0 statistical package (SPSS, Inc., Chicago, IL, USA). We used the chi-square test and Fisher’s exact test for categorical variables. The t-test or Mann–Whitney U test were used for continuous variables. The relationships of DFS and OS with the ALBI grade were analyzed using Kaplan–Meier survival curves and the log-rank test, and *p* < 0.05 was considered statistically significant. Factors that were significant in the univariate analysis (*p* < 0.05) were included in a multivariate analysis by using a Cox forward stepwise variable selection process of the estimated OS and DFS.

## Results

### Characteristics of the study population

Patient characteristics are shown in Table 1. There were 131 (78%) men and 37 (22%) women, with a mean age of 66 years at enrollment. 58 patients (34.5%) had diabetes mellitus (DM). The etiologies for NBNC-HCC were alcohol (19%), nonalcoholic fatty liver disease (NAFLD) (29.2%), followed by autoimmune hepatitis (AIH) (8.3%). The greater majority of patients (43.5%) has an unclassified etiology. Liver cirrhosis was observed in 38 patients (22.6%), and high preoperative alpha-fetoprotein (AFP) levels (> 200 ng/mL) were observed in 25 (15.3%) patients. The mean tumor size was 5.3 ± 3.7 cm, and four patients had multiple tumors. 66, 98, and 4 patients had ALBI grade I, II, and III, respectively. Because of the small number of patients in the ALBI grade III group, we combined patients with ALBI grades II and III for further analysis. Compared to patients with ALBI grade I, patients with ALBI grades II and III were significantly older (*p* = 0.002), had lower serum albumin levels (*p* < 0.001), and had a higher percentage of patients with Child–Pugh grade B (*p* = 0.009). There were no differences in the serum bilirubin level or tumor characteristics between the two groups.

**Table 1:** Comparison of clinical and pathological characteristics between patients with pre-operative ALBI grades I and II

	Total (n = 168)	ALBI grade I (n = 66)	ALBI grade II/III (n = 102)*	*p* value
Age (years; mean ± SD)	65.2 ± 12.2	58.9 ± 11.8	64.8 ± 12	0.002
Male, n (%)	131 (78%)	56 (84.8%)	75 (73.5%)	0.084
Diabetes mellitus, n (%)	58 (34.5%)	22 (33.3%)	36 (35.3%)	0.794
Hypertension, n (%)	90 (58.1%)	37 (60.7%)	53 (56.4%)	0.598
Etiology				0.066
Alcohol^$^, n (%)	32 (19.0%)	16 (24.2%)	16 (15.7%)	
NAFLD, n (%)	49 (29.2%)	24 (36.4%)	25 (24.5%)	
AIH, n (%)	14 (8.3%)	3 (4.5%)	11 (10.8%)	
Unclassified, n (%)	73 (43.5%)	23 (34.8%)	50 (49.0%)	
Current alcohol intake^$^, n (%)	32 (24.2%)	16 (32.7%)	16 (19.3%)	0.083
AST (U/L; mean ± SD)	41.6 ± 74.4	34.7 ± 19.3	46.1 ± 94.1	0.333
ALT (U/L; mean ± SD)	44.1 ± 109.4	39.5 ± 23.3	47.2 ± 139.3	0.658
Total bilirubin (mg/dL; mean ± SD)	0.8 ± 0.3	0.8 ± 0.3	0.8 ± 0.4	0.132
Albumin (g/dL; mean ± SD)	3.7 ± 0.6	4.2 ± 0.3	3.4 ± 0.4	< 0.001
Platelet (< 150,000 u/L), n (%)	38 (22.9%)	14 (21.5%)	24 (23.8%)	0.739
AFP (> 200 ng/mL), n (%)	25 (15.3%)	11 (16.9%)	14 (14.3%)	0.647
Liver cirrhosis, n (%)	38 (22.6%)	13 (19.7%)	25 (24.5%)	0.466
Tumor size (cm; mean ± SD)	5.3 ± 3.7	4.5 ± 2.9	5.9 ± 4.1	0.09
Tumor number (single:multiple)	164:4	65:1	99:3	0.554
Child–Pugh grade (A:B)	158:10	66:0	92:10	0.009
BCLC stage (0:A:B)	17:87:64	10:35:21	7:52:43	0.144
Pathological features				
Fat content (%) in nontumor tissue, n (%)**				0.064
> 30%	24 (17.5%)	15 (26.3%)	9 (11.3%)	
5–30%	37 (27.0%)	15 (26.3%)	22 (27.5%)	
< 5%	76 (55.5%)	27 (47.4%)	49 (61.3%)	
Microvascular invasion, n (%)	55 (35%)	19 (29.7%)	36 (38.7%)	0.224
pTNM stage (I:II:III)	101:58:8	41:23:1	60:3 5:7	0.289
Histological grade (well:moderate:poor)	31:125:11	14:50:2	17:75:9	0.365

*NAFLD* nonalcoholic fatty liver disease, *AIH* autoimmune hepatitis, *AST* Aspartate aminotransferase, *ALT* Alanine aminotransferase, *AFP* α-fetoprotein, *BCLC* Barcelona Clinic Liver Cancer, *ALBI* albumin–bilirubin, *pTNM* pathological tumor-node-metastasis

*4 cases are ALBI grade III

^$^ > 80 g ethanol per day

**137 cases had available data

### Survival analysis

**Fig. 2:**
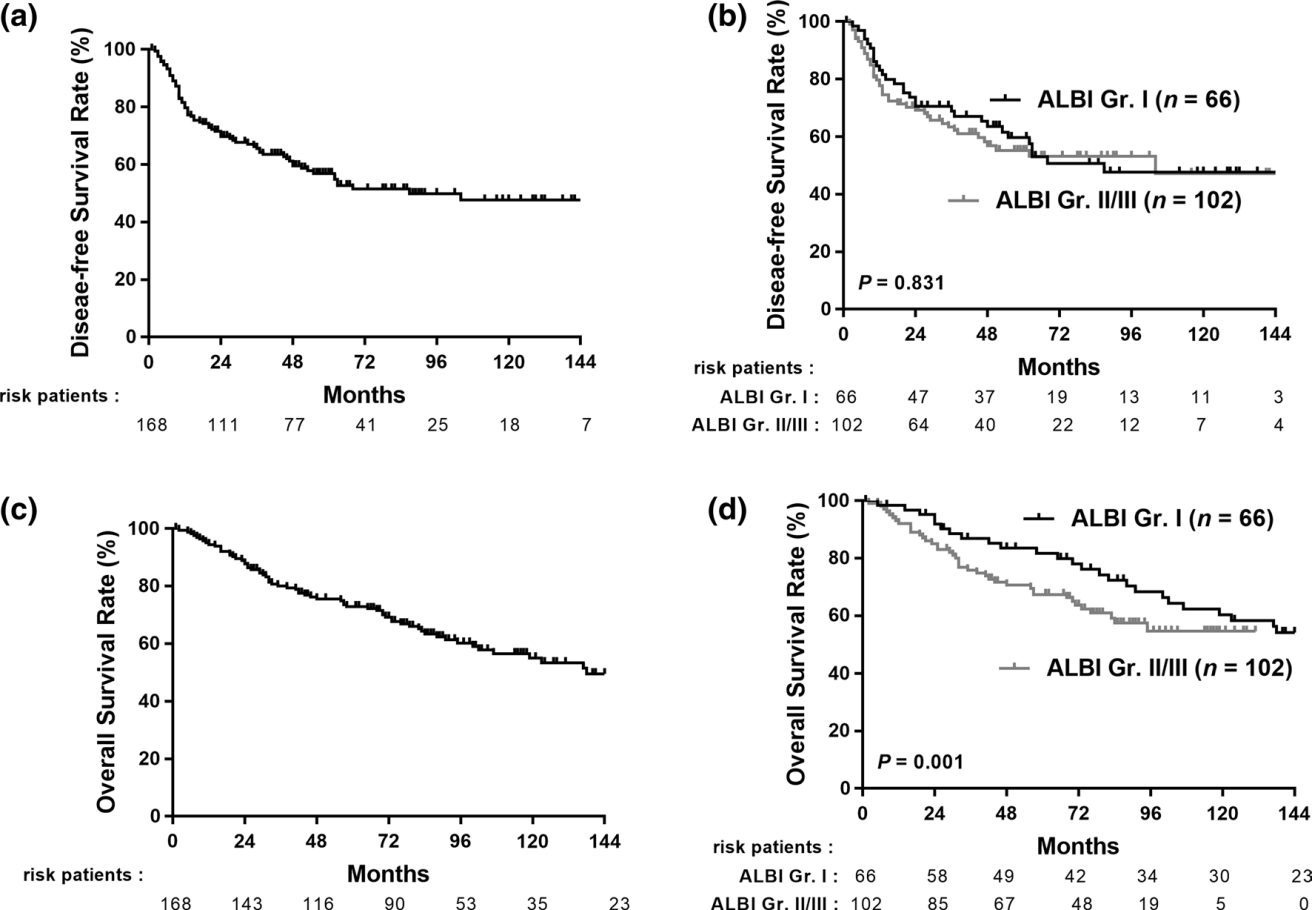
The disease-free survival (DFS) rate and overall survival (OS) rate in NBNC-HCC after resection. **A** DFS in the study population, **B** DFS between HCC patients with ALBI Gr I and Gr II/III, **C** OS in the study population, **D** OS between HCC patients with ALBI Gr I and Gr II/III

**Fig. 3:**
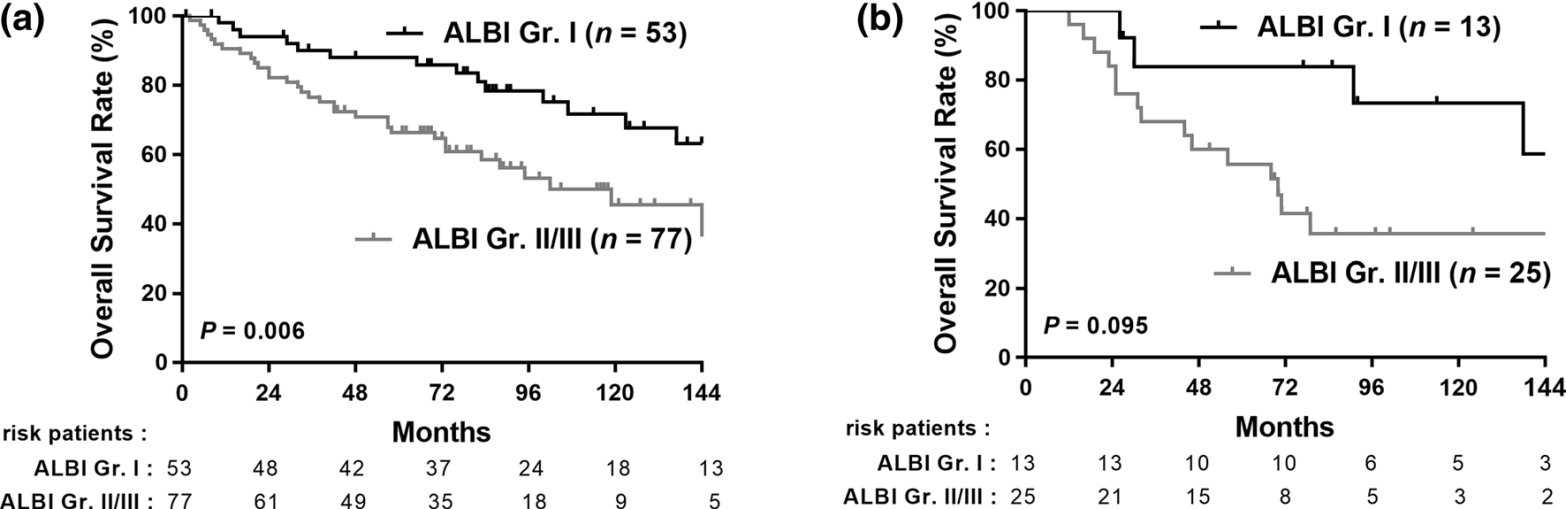
The cumulative overall survival in patients with NBNC-related HCC after curative resection among **A** non cirrhotic patients and **B** cirrhotic patients

After a median follow-up of 76 months, 74 patients (44%) developed recurrence, and 72 (42.9%) died. The 1-, 3-, and 5-year DFS rates were 80.4%, 66.3%, and 56.8%, respectively (Fig. 2A). There was no significant difference in the DFS between the ALBI grade I and ALBI grade II/III groups (*p* = 0.831, Fig. 2B). The 1-, 3-, and 5-year OS rates were 93.4%, 79.2%, and 72.0%, respectively (Fig. 2C). The ALBI grade I group had better OS than the ALBI grades II/III group (*p* = 0.001) (Fig. 2D). We further stratified by cirrhotic status (Fig. 3). NBNC-HCC patients with no cirrhosis and ALBI grade I had better OS than those with ALBI grades II/III (*p* = 0.006) (Fig. 3A). However, there was no significant difference in OS based on the ALBI grade among patients with cirrhosis (Fig. 3B).

### Independent factors for DFS and OS of NBNC-HCC patients after curative resection

Based on the multivariate Cox proportion hazards model, AFP > 200 ng/mL (hazard ratio [HR], 2.070, 95% CI 1.114–3.848, *p* = 0.021), number of tumors (HR, 10.770, 95% CI 2.513–46.153, *p* = 0.001) and pTNM stage (HR, 1.962, 95% CI 1.199–3.210*,* *p* = 0.007) were independent risk factors for HCC recurrence (Table 2). In OS analysis, the multivariate Cox proportional hazards model revealed that AFP > 200 ng/mL (HR, 2.729, 95% CI 1.452–5.130, *p* = 0.002), ALBI grade II/III (HR, 2.432, 95% CI 1.392–4.250, *p* = 0.002), and pTNM stage (HR, 4.902, 95% CI 2.036–11.803, *p* < 0.001) were independent risk factors for OS (Table 3). Child–Pugh grade was not an independent risk factor for OS after adjusting other factors in the multivariate analysis.

**Table 2:** Univariate and multivariate analysis of prognostics factors for recurrence in NBNC-HCC patients after curative resection

Variable	Comparison	Univariate		Multivariate	
		HR (95%CI)	*p* value	HR (95%CI)	*p* value
Age (years)	> 60 vs ≦ 60	1.316 (0.815–2.125)	0.216		
Sex	Male vs. Female	1.220 (0.698–2.131)	0.485		
DM history	Yes vs. no	1.286 (0.800–2.068)	0.299		
Hypertension	Yes vs. no	1.184 (0.694–2.020)	0.536		
Alcoholic history	Yes vs. no	1.353 (0.748–2.446)	0.318		
AFP (ng/ml)	> 200 vs. ≦ 200	2.065 (1.149–3.710)	0.015	2.070 (1.114–3.848)	0.021
Platelet (${10}^{9}$/L)	≦ 150 vs. > 150	1.264 (0.754–2.119)	0.375		
Albumin (g/dL)	≦ 3 vs. > 3	1.358 (0.833–2.215)	0.220		
Liver cirrhosis	Yes vs. no	0.950 (0.552–1.635)	0.853		
Child–Pugh grade	B vs. A	1.216 (0.682–2.168)	0.508		
ALBI grade	II/III vs. I	1.052 (0.662–1.671)	0.831		
Tumor size (cm)	> 5 vs. ≦ 5	1.024 (0.634–1.653)	0.923		
Tumor no	Multiple vs. single	2.847 (0.892–9.084)	0.077	10.770 (2.513–46.153)	0.001
BCLC stage	B vs. 0/A	1.004 (0.625–1.615)	0.985		
Liver fat content (%)	> 30 vs. ≦ 30	1.472 (0.811–2.673)	0.204		
Microvascular invasion	Yes vs. no	0.798 (0.479–1.329)	0.386		
pTNM stages	II + III vs. I	2.084 (1.302–3.336)	0.002	1.962 (1.199–3.210)	0.007
Histology stages	Poor vs. well/moderate	1.699 (0.682–4.232)	0.255		

*DM* diabetes mellitus, *AST* aspartate aminotransferase, *ALBI* albumin–bilirubin, *BCLC* Barcelona Clinic Liver Cancer, *pTNM* pathological tumor-node-metastasis

**Table 3:** Univariate and multivariate analysis of prognostics factors for overall survival in NBNC-HCC patients after curative resection

Variable	Comparison	Univariate		Multivariate	
		HR (95%CI)	*p* value	HR (95%CI)	*p* value
Age (years)	> 60 vs ≦ 60	1.971 (1.161–3.348)	0.012		
Sex	Male vs. Female	0.996 (0.569–1.641)	0.899		
DM	Yes vs. no	1.439 (0.894–2.315)	0.134		
Hypertension	Yes vs. no	1.099 (0.655–1.842)	0.721		
Alcoholic history	Yes vs. no	1.452 (0.785–2.687)	0.235		
AFP (ng/mL)	> 200 vs. ≦ 200	2.303 (1.248–4.251)	0.008	2.729 (1.452–5.130)	0.002
Platelet (${10}^{9}$/L)	≦ 150 vs. > 150	1.031 (0.596–1.785)	0.912		
Albumin(g/dL)	≦ 3 vs. > 3	2.369 (1.338–4.193)	0.003		
Liver cirrhosis	Yes vs. no	1.537 (0.919–2.568)	0.101		
Child–Pugh grade	B vs. A	1.545 (0.621–3.888)	0.346		
ALBI grade	II/III vs. I	3.395 (1.420–4.038)	0.001	2.432 (1.392–4.250)	0.002
Tumor size (cm)	> 5 vs. ≦ 5	1.481 (0.924–2.375)	0.103		
Tumor no	Multiple vs. Single	2.029 (0.636–6.475)	0.232		
BCLC stage	B vs. 0/A	1.456 (0.910–2.329)	0.117		
Liver fat content (%)	> 30 vs. ≦ 30	0.802 (0.376–1.711)	0.569		
Microvascular invasion	Yes vs. no	0.667 (0.389–1.145)	0.142		
pTNM stages	III vs. I + II	5.669 (2.543–12.640)	< 0.001	4.902 (2.036–11.803)	< 0.001
Histology stages	Poor vs. well/moderate	2.085 (0.832–5.230)	0.117		

*DM* diabetes mellitus, *AST* aspartate aminotransferase, *ALBI* albumin–bilirubin, *BCLC* Barcelona Clinic Liver Cancer, *pTNM* pathological tumor-node-metastasis

## Discussion

To the best of our knowledge, this is the first study to identify the preoperative ALBI grade as a useful prognostic marker of NBNC-HCC after curative resection. The OS rate at 5 years after curative resection in the ALBI grade I group was 87.2%, whereas it was only 62.4% in the ALBI grade II/III group. In the era of preventable strategies for HBV infection and curative treatments for HCV infection, the outcomes of NBNC-HCC deserve more attention. This study was a large-scale cohort study and demonstrated that a higher preoperative ALBI grade correlated with poor OS, but not with HCC recurrence, among NBNC-HCC patients after resection.

The Child–Pugh grading system is traditionally used for liver function assessment in patients with liver disease. It was created in the early 1970s as a method for prognostication of chronic liver disease [23]. Many HCC staging systems, such as the BCLC staging system, Cancer of the Liver Italian Program, and Japan integrated staging system, also integrate the Child–Pugh grade. However, ascites and hepatic encephalopathy, two of the five parameters in the Child–Pugh scoring evaluation, are dependent on physical examination and can be modified by medication. Recently, the ALBI score has been established for evaluating hepatic functional reserve. Multiple studies have proven the correlation of the ALBI score and prognosis after hepatectomy, radiofrequency ablation (RFA), transarterial chemoembolization (TACE), radiotherapy, and systemic therapy [24]. In contrast to the Child–Pugh grade, the ALBI grade uses only two objective serological markers, albumin and bilirubin, and subjective factors such as ascites and encephalopathy are not included. A growing number of clinical investigations suggested that the ALBI grade can more accurately predict the incidence of postoperative liver failure and OS than the Child–Pugh grade. In the present study, we also compared the areas under the curve (AUCs) for the Child–Pugh score and ALBI grade in predicting postoperative survival, and the result showed that the ALBI grade had a higher AUC than the Child–Pugh score (0.641 vs. 0.494) (Additional file 1: Figure S1). This result might be because that patients in the same Child–Pugh classification could be separated into different ALBI grade and have survival difference with the wide range of hepatic reserve within a single Child–Pugh classification. Furthermore, the evaluation of ascites and encephalopathy is highly subjective and may greatly reduce the accuracy of the assessment. In the present study, the majority (94%) of patients were classified into Child–Pugh class A. In contrast, patients with Child–Pugh class A could further divided 66 (41.8%) into ALBI grade I and 92 (58.2%) into ALBI grade II, which were significant different in OS (*p* = 0.001).

Although HBV and HCV are still the leading causes of HCC, the incidence of NBNC-HCC has been increasing in Taiwan [21]; consequently, the rates of resected NBNC-HCC also increased in our cohort over time. The recruitment also increased over time, with 13% of patients being recruited in 2001–2005, 29% in 2006–2010, and 58% in 2011–2016. Our data imply that NBNC-HCC should not be overlooked, although the present study did not have a control arm for patients with viral hepatitis.

Patients with NBNC-HCC in this study had a higher proportion of DM than cohorts in our previous studies [19, 25]. This result was similar to that in a large cohort study in Taiwan, which enrolled 3843 patients with HCC from The Taiwan Liver Cancer Network [7]. Huang et al. investigated 411 patients with NBNC-HCC, 420 matched patients with HBV-HCC, and 420 matched patients with HCV-HCC, and the highest prevalence (33%) of DM was found in the NBNC-HCC cohort. In addition, the degrees of fatty change in the liver tissue in our cohort were similar to those in Huang’s study, and fatty liver was significantly more common in patients with NBNC-HCC than in patients with HBV-HCC or HCV-HCC. Our results confirmed that metabolic risk factors were associated with patients with NBNC-HCC.

In addition to the ALBI grade, we also found that serum AFP, number of tumors, and pTNM stage were independent risk factors for HCC recurrence. Furthermore, age, AFP, multiple tumors, and pTNM stage were risk factors for OS. These results were similar to those of previous studies [19, 26, 27] but not identical, which may be because of differences in the HCC population. In the present study, we focused on NBNC-HCC patients after curative resection, whereas previous studies focused on HCC related to HBV, HCV, or both. The underlying mechanism of HCC from non-viral hepatitis may be different from that of HCC from viral hepatitis. However, we believe that the preoperative ALBI grade is a useful marker for predicting the outcomes of HCC patients after curative resection, regardless of whether patients have HBV-, HCV-, or NBNC-related HCC.

We did not have data regarding occult hepatitis B infection (OBI). Several epidemiological and molecular studies have reported that OBI plays an important role in the progression of cirrhosis and the development of HCC. OBI is defined as the presence of replication-competent HBV DNA in the liver and/or HBV DNA in the blood in an individual with serum HBsAg negativity assessed by currently available assays [28]. OBI is the combined result of host immune control and different genomic expressions of the virus. It leads to a virological quiescent state; hence, the vast majority of OBI cases have low levels of HBV DNA [29]. The prevalence of OBI varies regionally worldwide and across patient populations, and higher rates have been reported in Asia than elsewhere [30]. In Taiwan, the prevalences are 0.11% in blood donors [31] and 10.9% in HBV-vaccinated children [32], and in patients with HBsAg-negative HCC, the prevalence of OBI may be higher. A meta-analysis showed an increased risk of HCC in patients with OBI in both retrospective (OR 6.06) and prospective studies (OR 2.86) [33]. Although most patients in our study did not have serum HBV DNA data to evaluate OBI, the result of the ALBI grade predicting the outcomes of NBNC-HCC after resection was unchanged.

In the present study, 74 patients developed recurrence. To treat that recurrence, 5 patients received hepatectomy, 25 received RFA, 34 underwent TACE, 2 received percutaneous ethanol injection, 4 received systemic treatment (targeted therapy or palliative chemotherapy), and 4 chose hospice care. In terms of OS, patients who received resection or RFA had better outcomes than patients who received TACE, followed by those who received systemic treatment or hospice care. Treatment of recurrence is based on age, performance status, tumor size, tumor number, lymph node involvement, and liver function reserve at recurrence. Therefore, regular follow-up and earlier detection of recurrence to ensure patients have preserved liver function can improve treatment and outcomes.

Some recent studies have demonstrated that postoperative markers, including the ALBI and platelet–albumin–bilirubin score, can predict the outcomes of HCC after resection [19, 34, 35]. This is because the postoperative ALBI grade can reflect the remnant liver, whereas the preoperative ALBI grade is affected by the tumor burden. We also tried to collect postoperative data, including albumin and bilirubin levels, to calculate the postoperative ALBI grade and albumin–bilirubin change; however, most data were missing or at different time points, making analysis difficult. In the future, a prospective study is needed to assess serial serum data, including platelets, albumin, and bilirubin, in order to evaluate the best time point of the ALBI grade to predict HCC outcomes.

We acknowledge the following limitations. First, this study was a single-center retrospective study. We did not collect data on intraoperative blood loss, amount of fluid received, blood transfusion, and the volume of the remnant liver, although a previous study showed that these data were not statistically significant [36]. Second, this was retrospective data from medical records and some data were missing, such as the postoperative albumin and bilirubin levels, which could have improved our outcome prediction. Therefore, a prospective study is needed for further assessment on the precise time to assess the ALBI grade to best predict prognosis. Although all of our patients were HBsAg-negative, we could not collect data on hepatitis core antibodies (anti-HBc) to exclude possible occult or past HBV infections. Occult hepatitis viral infection should be considered because Taiwan is an endemic area for HBV infection, and the prevalence of anti‐HBc may be high for those born before universal vaccination was instituted. In the future, the complete analysis, including the anti-HBc, hepatitis B surface antibody, and HBV DNA statuses, was noteworthy to clarify the role of OBI in these special populations.

## Conclusions

In conclusion, the ALBI grade may predict OS in NBNC-HCC patients after curative resection. Although there was no significant difference in HCC recurrence based on the ALBI grade, we still observed that patients with ALBI grade II/III had poorer DFS than those with ALBI grade I. Hence, we believe that the ALBI grade is a promising noninvasive marker for predicting the outcomes of NBNC-related HCC patients after curative resection.

## Supplementary Information

**Additional file 1: Figure S1.** Comparisons of the areas under the curve (AUC) between ALBI grade and Child–Pugh class for outcome predictions in NBNC-HCC patients after operations.

## Data Availability

All analyzed data are included in this published article. The original data are available upon reasonable request to the corresponding author.

## Abbreviations

NBNC: Non-B non-C
HCC: Hepatocellular carcinoma
BCLC: Barcelona Clinic Liver Cancer
OS: Overall survival
DFS: Disease-free survival
HR: Hazard ratio
HBV: Hepatitis B virus
AFP: Alpha-fetoprotein
HBsAg: Hepatitis B surface antigen
anti-HBc: Hepatitis B core antibodies
ALBI: Albumin–bilirubin
OBI: Occult hepatitis B infection
