# Supplementary figures and images for: Preoperative ALBI grade predicts the outcomes in non-B non-C HCC patients undergoing primary curative resection
Source: BMC Gastroenterol. 2021 Oct 19;21:386. doi: 10.1186/s12876-021-01944-w (PMC8524867; doi:10.1186/s12876-021-01944-w)

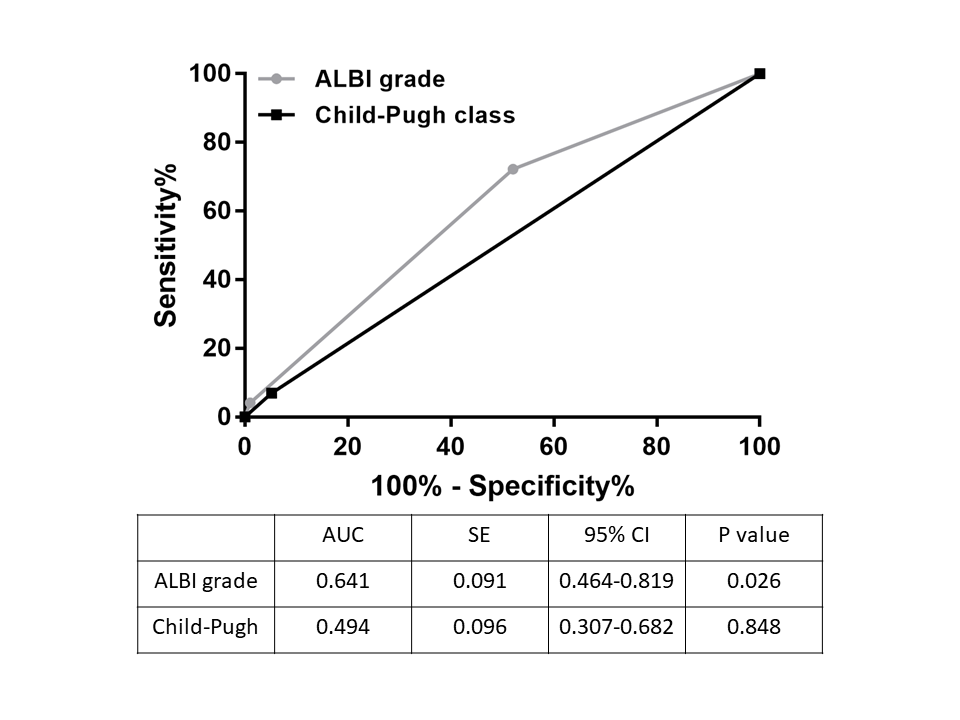

Supplement: Supplementary file 1 — Additional file 1: Figure S1. Comparisons of the areas under the curve (AUC) between ALBI grade and Child–Pugh class for outcome predictions in NBNC-HCC patients after operations. [file 12876_2021_1944_MOESM1_ESM.tiff]
